# Supplementary material for: LHW-Net: An ensemble-based machine learning framework for brain tumor classification
Source: PLoS One. 2026 Apr 21;21(4):e0346821. doi: 10.1371/journal.pone.0346821 (PMC13098932; doi:10.1371/journal.pone.0346821)
Supplement: S1 File — (PDF) [file pone.0346821.s001.pdf]

S1 File. Description of the datasets used in this study, with download links.

**Dataset Name**	**Link**
Brain Tumor MRI Dataset:	https://www.kaggle.com/datasets/masoudnickparvar/brain-tumor-mri-dataset
Brain Tumor Image Dataset	https://www.kaggle.com/datasets/denizkavi1/brain-tumor/data
Br35H Brain Tumor Detection 2020	https://www.kaggle.com/datasets/ahmedhamada0/brain-tumor-detection
