# Supplementary material for: LHW-Net: An ensemble-based machine learning framework for brain tumor classification
Source: PLoS One. 2026 Apr 21;21(4):e0346821. doi: 10.1371/journal.pone.0346821 (PMC13098932; doi:10.1371/journal.pone.0346821)
Supplement: Attachment — Submitted filename: Response to Reviewers.docx [file pone.0346821.s003.docx]

**Reviewers’ Comments, Author Responses**

**Title: LHW-Net: An Ensemble-Based Machine Learning Framework for Brain Tumor Classification**

**Authors: Thireesha Suryadevara, Naveenkumar Mahamkali, Mudassir Rafi**

**Journal: PLOS ONE**

Dear Reviewer (s),

We sincerely thank you for your time and effort in reviewing our manuscript. Your detailed and constructive feedback has been invaluable in improving the clarity, rigor, and overall quality of the work. We have carefully addressed each of your comments and incorporated your suggestions into the revised version.

**Reviewer 1:**

| **Comment 1:**  The quality of the figures is low. Please consider providing the images in higher resolution, as the information in the images is difficult to read. | **Response:**  The figures have been replaced with more detailed ones (300 dpi) to make them clear and readable. Moreover, the subfigures are eliminated and rearranged as separate figures to present them better. The revised version of the manuscript consists of clearer plots, confusion matrices, and sample images to visualize them better.  **Location in Paper:** Throughout the paper. |
| --- | --- |
| **Comment 2:**  Did the authors split the data into training and testing sets after data augmentation? I am concerned about potential data leakage if this approach was used. | **Response:** In the revised version, we ensured that no data leakage occurred. For Dataset I, the data was already provided as predefined training and test sets, and only the training set was augmented. For Datasets II and III, the data was first divided into training and test sets, after which only the training sets were augmented.  The overall process of data splitting and augmentation has been clearly illustrated in Figures 3 and 5 of the revised manuscript.    **Location in Paper:** Page numbers: 05, Line number 180 and 181. |
| **Comment 3:**  Could you double check if this CUDA version is correct: CUDA 12.5555.42.02? | **Response:** Thank you for your suggestion. The CUDA 12.5 version is supported by the driver, whereas our experiments were based on TensorFlow 2.4.0 and CUDA 11.2, cuDNN 8. Manuscript was updated to eliminate confusion.  **Location in Paper:** Page numbers: 11, Line number 345 to 350. |
| **Comment 4:**  The part of "Ablation Study for Base Learner Identification" should be moved to the section of "Experimental Results and Discussion", as it presents the findings of your experiment. | **Response:** As per your suggestion the manuscript had restructured accordingly.  **Location in Paper:** Page numbers: 11 and 12. |
| **Comment 5:**  The "Performance measures" should be moved to the section of "Materials and Methods", as it describes the evaluation metrics used to measure performance. | **Response:** As per your suggestion the manuscript had restructured accordingly.  **Location in Paper:** Page numbers: 10. |
| **Comment 6:**  In Table 7, please specify the classifier that produced the optimal outcomes in your proposed model, rather than writing “Combination of Feature Fusion (LBP, HOG, Wavelet) with Various Classifiers.” | **Response:** Table 7 has been updated to include a specific reference to the classifier(s) that obtained the best results on each dataset (e.g. LBP+SVC, HOG+KNN, Wavelet+SVC) .  **Location in Paper:** Page numbers: 15. |
| **Comment 7:** I am a bit confused about the design of the proposed model. Is it an ensemble model with three classifiers at the last layer, or is it three individual models, each with one classifier (KNN, RF, SVC)? | **Response:** In the initial stage, we employed three different classifiers—KNN, RF, and SVC—to train individually on distinct feature sets (LBP, HOG, and Wavelet, respectively). We then evaluated multiple feature–classifier pairings through probabilistic score fusion. The most effective combinations identified were LBP + SVC, HOG + KNN, and Wavelet + SVC, which were subsequently integrated at the decision level.  Therefore, the proposed approach does not consist of three independent models but represents a single ensemble framework, wherein feature-specific classifiers contribute to a unified final decision.  **Location in Paper:** This clarification has been added to the revised manuscript on Page 14, Lines 421–424. |
| **Comment 8:**  What are the limitations of the experiment? This should be mentioned in the section of "Experimental Results and Discussion". | **Response:** In the revised manuscript, we have explicitly mentioned the limitations in the “Experimental Results and Discussion” section. The current framework demonstrates promising accuracy; however, it has the following limitations:   1. The proposed model relies on handcrafted feature descriptors, which may not capture deeper semantic representations inherent in complex attack patterns. 2. The approach is computationally intensive, particularly when using the SVC classifier with large feature sets, leading to increased processing time.   These limitations have been discussed along with potential directions for improvement in future work.  **Location in Paper:** The corresponding details are provided on Page 14, Lines 442–448 of the revised manuscript. |

**Response to Editorial Requirements:**

| **Comment 1:**  Please ensure that your manuscript meets PLOS ONE's style requirements, including those for file naming. | **Response:** We have modified the manuscript according to the PLOS ONE LaTeX template, to ensure that it is presented according to the style standards of the journal such as file names and formatting. |
| --- | --- |
| **Comment 2:**  Please note that PLOS One has specific guidelines on code sharing for submissions in which author-generated code underpins the findings in the manuscript. In these cases, we expect all author-generated code to be made available without restrictions upon publication of the work. | **Response: T**he author-created code of this study is stored in the following GitHub repository: (<https://github.com/thireesha-suryadevara/LHW-Net>). |
| **Comment 3:**  Thank you for stating the following financial disclosure: The authors extend their appreciation to the Deanship of Research and Graduate Studies at King Khalid University for funding this work through Large Research Project under grant number RGP2/588/46.  Please state what role the funders took in the study. If the funders had no role, please state: "The funders had no role in study design, data collection and analysis, decision to publish, or preparation of the manuscript." If this statement is not correct you must amend it as needed. Please include this amended Role of Funder statement in your cover letter; we will change the online submission form on your behalf. | **Response:** The author Dr. Mudassir Rafi is having dual affiliation as mentioned in the manuscript. Currently, he is associated with the funder, King Khalid University, as an Assistant Professor and as one of the supervisors for the first author, Mrs Thireesha Suryadevara. He is involved in Supervision, reviewing, advising and fund acquisition for the submitted work as stated in the financial disclosure. |
| **Comment 4:**  When completing the data availability statement of the submission form, you indicated that you will make your data available on acceptance. We strongly recommend all authors decide on a data sharing plan before acceptance, as the process can be lengthy and hold up publication timelines. Please note that, though access restrictions are acceptable now, your entire data will need to be made freely accessible if your manuscript is accepted for publication. This policy applies to all data except where public deposition would breach compliance with the protocol approved by your research ethics board. If you are unable to adhere to our open data policy, please kindly revise your statement to explain your reasoning and we will seek the editor's input on an exemption. Please be assured that, once you have provided your new statement, the assessment of your exemption will not hold up the peer review process. | **Response:** We had updated the Data Availability Statement to reflect our intended plan for sharing the data. The data used in this study is publicly available on Kaggle.  All code created by the authors (programming code) has been placed in (<https://github.com/thireesha-suryadevara/LHW-Net>) githhub repository. |
| **Comment 5:**   Please upload a new copy of Figures 4 and 6 as the detail is not clear. | **Response:** Figures 4 and 6 are also revised to higher-resolution images (at least 300 dpi) and the subfigures have been replaced to individual full-resolution images, which improves quality and visibility of details. The workflow is represented in figures 3, 5, 7, 8, and 9 of the revised manuscript. |
| **Comment 6:**  Please include captions for your Supporting Information files at the end of your manuscript, and update any in-text citations to match accordingly. | **Response:** We included captions for all Supporting Information files at the end of the manuscript, while independently ensuring in-text citations matched the respective files. |
| **Comment 7:**  If the reviewer comments include a recommendation to cite specific previously published works, please review and evaluate these publications to determine whether they are relevant and should be cited. There is no requirement to cite these works unless the editor has indicated otherwise. | **Response:** We thank the editor for this clarification. Upon reviewing all reviewer comments, we found that no additional citations were specifically recommended for inclusion. Therefore, no new references have been added in this revision. |
