# Supplementary material for: LHW-Net: An ensemble-based machine learning framework for brain tumor classification
Source: PLoS One. 2026 Apr 21;21(4):e0346821. doi: 10.1371/journal.pone.0346821 (PMC13098932; doi:10.1371/journal.pone.0346821)
Supplement: Attachment — Submitted filename: Response_to_Reviewers_auresp_2.docx [file pone.0346821.s004.docx]

**LHW-Net: An Ensemble-Based Machine Learning Framework for Brain Tumor Classification**

Dear Editor and Reviewers,

Thanks to your time, effort and insightful feedback on our manuscript. Your critical and detailed remarks have played a major role in the refining of our work. We have taken note of every point and used your suggestions in the revised version.

**Reviewer 1:**

| **Comment 1:**  Please don’t use yellow as a highlight color for revisions. It is difficult to read. | **Response:**  In the revised manuscript, the yellow highlight has been changed to a more readable color (blue) to enhance clarity.  **Location in Paper:** Throughout the paper. |
| --- | --- |
| **Comment 2:**  For the figures, I still think the quality of images should be enhanced. | **Response:**  Thank you for the comment. The figures have been improved in terms of resolution, font sizes, and overall clarity to improve their readability. All figures were checked with NewGen ARTANALYSIS (NAAS tool) in order to match the requirements of the journal in terms of formatting and presentation.  **Location in Paper:** Throughout the paper. |

**Reviewer 2:**

| **Comment 1:**  Improve resolution and clarity of all figures and increase font size in diagrams and confusion matrices. | **Response:**  Every figure has been updated with the improvement of their resolution, the enlargement of fonts in the diagrams and confusion matrices, and the overall clarity to make them easier to read and present better. All figures were checked with NewGen ARTANALYSIS (NAAS tool) in order to match the requirements of the journal in terms of formatting and presentation.  **Location in Paper:** Throughout the paper. |
| --- | --- |
| **Comment 2:**  Clarify preprocessing steps (resizing, normalization, etc.). | **Response:**  We have revised the paper to explain the preprocessing steps, such as Gray scale conversion, resizing etc.  **Location in Paper:** Page number: 05, Line number 154 to 187. |
| **Comment 3:**  Explain hyperparameter tuning strategy. | **Response:**  We have provided the description of the hyperparameter tuning strategy in the revised manuscript. The three models, which are KNN, Random Forest, and SVM, were tested on three datasets with varying hyperparameter settings. As the performance varied with different parameter settings, the optimal hyperparameters were empirically determined based on the best overall performance across the datasets. The selected parameters are: KNN (*n_neighbors=3*), Random Forest, (*n_estimators=100*), and Linear SVM.  **Location in Paper:** Page number: 08, Line number 274 to 290. |
| **Comment 4:**  Fix minor typos and formatting inconsistencies. | **Response:**  The paper has been carefully checked, and all minor typographical errors and formatting discrepancies have been removed.  **Location in Paper:** Throughout the paper. |

**Response to Editorial Requirements:**

| **Comment 1:**   If the reviewer comments include a recommendation to cite specific previously published works, please review and evaluate these publications to determine whether they are relevant and should be cited. There is no requirement to cite these works unless the editor has indicated otherwise. | **Response:**  The reviewer did not make any specific previously published works to be cited. But we have reviewed the literature attentively and included a few recent and relevant references to make the manuscript more powerful and justified.  **Location in Paper:** Page number: 15. |
| --- | --- |
| **Comment 2:**   Please review your reference list to ensure that it is complete and correct. If you have cited papers that have been retracted, please include the rationale for doing so in the manuscript text, or remove these references and replace them with relevant current references. Any changes to the reference list should be mentioned in the rebuttal letter that accompanies your revised manuscript. If you need to cite a retracted article, indicate the article’s retracted status in the References list and also include a citation and full reference for the retraction notice. | **Response:**  The reference list has been carefully reviewed to make sure that every entry is complete and correct. No retracted articles are mentioned. Moreover, some recent and relevant publications were added to further justify and prove the proposed work. The changes in the reference list have been added to the revised manuscript.  **Location in Paper:** Page number: 15. |
